# Long-term outcomes of prostate intensity-modulated radiation therapy incorporating a simultaneous intra-prostatic MRI-directed boost

**DOI:** 10.3389/fonc.2022.921465

**Published:** 2022-08-10

**Authors:** Brady S. Laughlin, Alvin C. Silva, Sujay A. Vora, Sameer R. Keole, William W. Wong, Michael H. Schild, Steven E. Schild

**Affiliations:** ^1^ Department of Radiation Oncology, Mayo Clinic, Phoenix, AZ, United States; ^2^ Department of Radiology, Mayo Clinic, Phoenix, AZ, United States; ^3^ Pinehurst Pathology Center, Pinehurst, NC, United States

**Keywords:** SIB, prostate cancer, IMRT (intensity modulated radiation therapy), radiation therapy, MRI

## Abstract

**Purpose/objectives:**

This retrospective study demonstrates the long-term outcomes of treating prostate cancer using intensity modulated (IMRT) with incorporation of MRI-directed boost.

**Materials/methods:**

From February 2009 to February 2013, 78 men received image-guided IMRT delivering 77.4 Gy in 44 fractions with simultaneously integrated boost to 81–83 Gy to an MRI-identified lesion. Patients with intermediate-risk or high-risk prostate cancer were recommended to receive 6 and 24–36 months of adjuvant hormonal therapy, respectively.

**Results:**

Median follow-up was 113 months (11–147). There were 18 low-risk, 43 intermediate-risk, and 17 high-risk patients per NCCN risk stratification included in this study. Adjuvant hormonal therapy was utilized in 32 patients (41%). The 10-year biochemical control rate for all patients was 77%. The 10-year biochemical control rates for low-risk, intermediate-risk, and high-risk diseases were 94%, 81%, and 88%, respectively (p = 0.35). The 10-year rates of local control, distant control, and survival were 99%, 88%, and 66%, respectively. Of 25 patients who died, only four (5%) died of prostate cancer. On univariate analysis, T-category and pretreatment PSA level were associated with distant failure rate (p = 0.02). There was no grade =3 genitourinary and gastrointestinal toxicities that persisted at the last follow-up.

**Conclusions:**

This study demonstrated the long-term efficacy of using MRI to define an intra-prostatic lesion for SIB to 81–83Gy while treating the entire prostate gland to 77.4 Gy with IMRT. Our study confirms that modern MRI can be used to locally intensify dose to prostate tumors providing high long-term disease control while maintaining favorable long-term toxicity.

## Background

Dose escalation has been a critical topic of investigation in the management of prostate cancer with radiotherapy (1–8). The entire prostate gland may be safely and effectively treated up to 80 Gy without increasing toxicity (3–6).

Studies evaluating patterns of failure following conventionally fractionated radiation demonstrated that 90% of local recurrences typically occur in the intraprostatic dominant nodule (9–12). The intraprostatic lesion is the largest nodule within the prostate which typically has the most aggressive behavior (13). Therefore, a strategy to boost these lesions with a higher dose to enhance the therapeutic ratio while limiting the dose to surrounding normal tissue was proposed (14).

Multiparametric MRI uses various T1 and T2 sequences, dynamic contrast enhancement, and diffusion-weighted imaging to identify prostate cancer versus normal prostatic tissue (15–17). Using the information from MRI scan for treatment planning, an escalated dose of radiation can be delivered to the dominant intraprostatic lesion. The advent of image-guided therapy and fiducial markers for tracking prostate movement has allowed the use of smaller margins minimizing the rectum and bladder within the high-dose region (18).

Herein, we report the long-term results of patients receiving a simultaneous integrated boost up to 83 Gy to intraprostatic lesions identified by MRI. This treatment strategy was devised in order to increase the therapeutic ratio by delivering radiation therapy focally to the region of prostate cancer. While there are many studies looking at the safety and short-term outcomes of simultaneous integrated boost to intraprostatic lesions using MRI, this is one of the few studies to present data with substantially longer follow-up.

## Materials and methods

Institutional review board approval was obtained prior to performing this retrospective analysis. Patients treated between February 2009 and February 2013 at our institution with MRI-guided boost for prostate cancer were analyzed in this study.

### Treatment planning and delivery

A multiparametric MRI was used to identify the intra-prostatic lesion (IPL) for treatment planning. T2-weighted, diffusion-weighted, and dynamic contrast-enhanced (DCE) images were obtained to define the IPL. A genitourinary diagnostic radiologist identified prostate lesions. Following the MRI, four fiducial markers were placed in the prostate. Patients were then simulated with computed tomography (CT) in the treatment position. The IPL was designated on MRI within the planning system.

Volume and organs at risk (OAR) definition and treatment planning were performed in Eclipse (Varian Medical Systems). The rectum and bladder were contoured as solid organs. The prostate was contoured to generate a low-dose target volume. The IPL was contoured to create the high-dose target volume. In patients with multiple IPLs, each IPL was included in the high-dose target volume. If an IPL was not identified on MRI, the region of biopsy positivity was contoured to create the high-dose target volume. The prostate volume was expanded by 3 mm to create the PTV for the lower dose with no expansion used for the SIB high-dose volume. The PTV low-dose volume (prostate plus 3 mm) received 77.4 Gy in 1.8 fractions with a simultaneous integrated boost of 83 Gy delivered to the PTV high-dose volume. Patients without an IPL on MRI had 81 Gy delivered to the PTV high-dose volume. Seminal vesicles received 75–77.4 Gy if involved and only 54 Gy if not involved. The adjacent dose-limiting normal structures including bladder, rectum, and femoral heads were contoured as organs at risk. This was our standard technique at that time based in large part on our experience performing a prospective trial utilizing IMRT and ProstaScint-based SIB (19).

Normal tissue dose constraints were as follows: ≤30% of the rectum or bladder could receive ≥70 Gy; ≤10% of the rectum or bladder could receive ≥75 Gy; and ≤1.8 cm^3^ of the rectum or bladder could receive ≥81 Gy.

Radiotherapy was delivered using intensity-modulated radiotherapy techniques (IMRT). IMRT plans were generated using seven-field IMRT, nine-field IMRT, or volumetric modulated arc therapy (VMAT-rotational IMRT) using 6- or 18-MV x-rays. Image guidance using kV matching of the four implanted fiducial markers was performed daily to localize the prostate.

Androgen deprivation therapy (ADT) was administered at the discretion of the treating physician and was recommended for intermediate- and high-risk diseases. Patients in the intermediate-risk group were advised to receive 6 months of ADT (leuprolide), and patients in the high-risk group were advised to receive 24–36 months of ADT. After radiotherapy, patients were evaluated at 3–12-month intervals with serum PSA measurement, physical examination, and toxicity assessments.

### Endpoints

Treatment outcomes were defined in terms of biochemical control (BC), overall survival (OS), local control (LC), and distant control (DC) rates. Length of follow-up and overall survival was determined as the time from completion of all treatment to date of last follow-up or death. Biochemical failure was defined as the time from end of radiation treatment to biochemical relapse defined per the American Society for Radiation Oncology-Phoenix definition (20). Local failure was defined as the time from end of radiation treatment to development of palpable or biopsy positive relapse within the prostate. Gastrointestinal (GI) and genitourinary (GU) side effects were graded using Common Terminology Criteria for Adverse Events (CTCAEv.4). Acute toxicity was defined as occurring during and within 3 months of radiotherapy. Chronic toxicity was considered as any time beyond 3 months from radiotherapy. Descriptive statistics were generated to determine baseline characteristics pertaining to diagnosis, treatment, outcomes, and toxicity. The Kaplan–Meier method was used to estimate BC, LC, DC, and OS rates. Univariate analysis (log-rank test) comparisons were performed with JMP software, version 14 (SAS Institute, Cary, NC).

## Results

### Treatment characteristics

Seventy-eight patients with clinical T1–3, N0 M0 prostate cancers were treated with conventional fractionated radiotherapy to the prostate gland. **Table 1** summarizes patient characteristics. The median age at start of IMRT was 76 years (range: 60 to 89 years). ADT was given to 32 patients (41%). Prior to delivery of radiotherapy, 77 (99%) patients underwent 1.5-Tesla (T) MP-MRI while 1 (1%) underwent 3-T MP-MRI. The median time from MRI to radiation treatment start was 20 days. Sixty-two (79%) patients received a dose of 83 Gy to one IPL. Sixteen (21%) of 78 patients had no identifiable IPL and therefore were treated with 81 Gy to areas of biopsy positivity. Treatment was delivered with seven-field IMRT, nine-field IMRT, or volumetric modulated arc therapy (VMAT) in 63 (81%), 7 (9%), and 8 (10%) patients, respectively.

**Table 1:** Patient characteristics and 10-year outcomes.

	n (%)	Biochemical control		Local control		Distant control		Overall survival	
		10-year value, % (95% CI)	*P* value*	10-year value, % (95% CI)	*P* value*	10-year value, % (95% CI)	*P* value*	10-year value, % (95% CI)	*P* value*
All patients	78 (100)	77 (74-80)		99 (-)		88 (85-91)		66 (62-70)	
T category			0.004		0.53		<0.001		0.54
1c	28 (36)	86 (80–92)		100		89 (87–91)		71 (65–77)	
2a	20 (26)	90 (85-95)		95		95		60 (52-68)	
2b	16 (20)	88 (83–93)		100		94		63 (53-73)	
2c	13 (17)	85 (76-94)		100		85 (75-95)		85(82-88)	
3a	1 (1)	0		100		0		0	
PSA level			.08		.04		0.02		0.63
≤10 ng/ml	60 (77)	88 (84–92)		100		93 (92-94)		70 (65–75)	
10-20 ng/ml	14 (18)	71 (58–84)		93		71 (59-83)		57 (47–67)	
>20 ng/ml	4 (5)	100		100		100		75	
Gleason score			.95		.81		0.90		0.43
6	26 (33)	88 (84–92)		100		92		73 (68–78)	
7	39 (50)	85 (80-90)		97		90 (85–95)		72 (66–78)	
8	12 (16)	83 (77–89)		100		83 (77–88)		50 (45–55)	
9	1 (1)	100		100		100		0	
NCCN risk group			0.35		0.65		0.80		0.53
Low	18 (23)	94		100		94		72 (66-78)	
Intermediate	43 (55)	81(76-86)		98		88 (83–93)		72 (66-78)	
High	17 (22)	88 (84–92)		100		88 (85–91)		53(49–57)	
Perineural invasion			0.36		0.10		0.13		0.95
No	56 (72)	88 (84-92)		100		93 (92–94)		69 (64–74)	
Yes	22 (28)	82 (75–89)		96		82 (75-89)		68 (60–76)	
Boost volume as a percentage of prostate volume			0.71		0.45		0.25		0.93
≤10%	68 (87)	87 (84–90)		99		91 (88–94)		68 (64-72)	
>10%	10 (13)	80 (73–87)		100		80 (66–94)		70 (56 – 84)	
% biopsy core positive			0.85				0.39		0.40
≤50	63 (81)	89(86–92)		100		93 (92–94)		65 (61–69)	
>50	15 (19)	87(78-96)		100		87 (79–95)		80 (72–88)	
Hormonal treatment			0.65		0.22		0.67		0.24
No	46 (59)	85 (80–90)		100		91 (90-92)		74 (69-79)	
Yes	32 (41)	88 (83-93)		97		88(83–93)		59 (53-65)	

*Univariate, log rank.

### Outcomes

**Figure 1:**
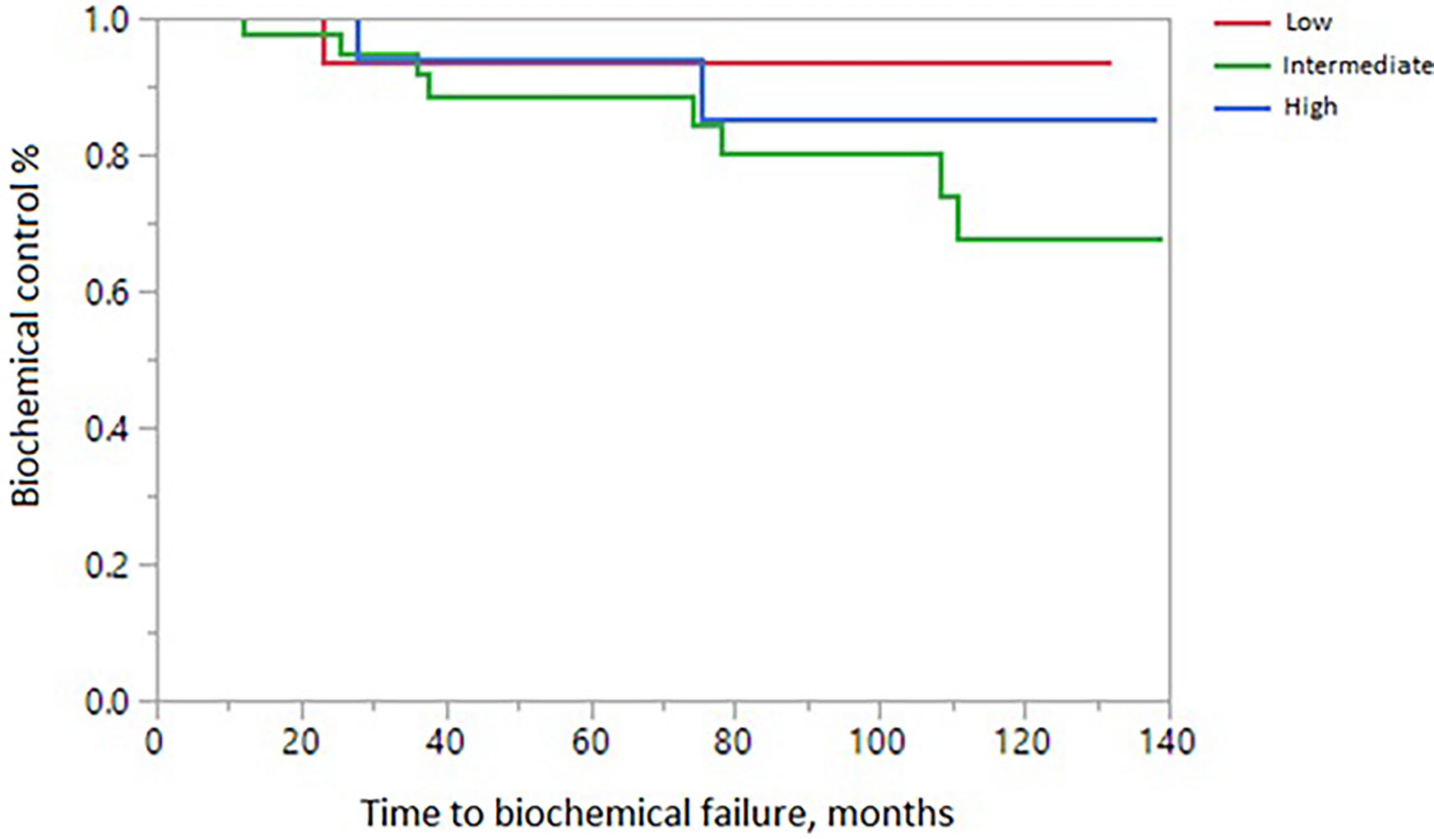
Biochemical control according to NCCN risk stratification.

**Figure 2:**
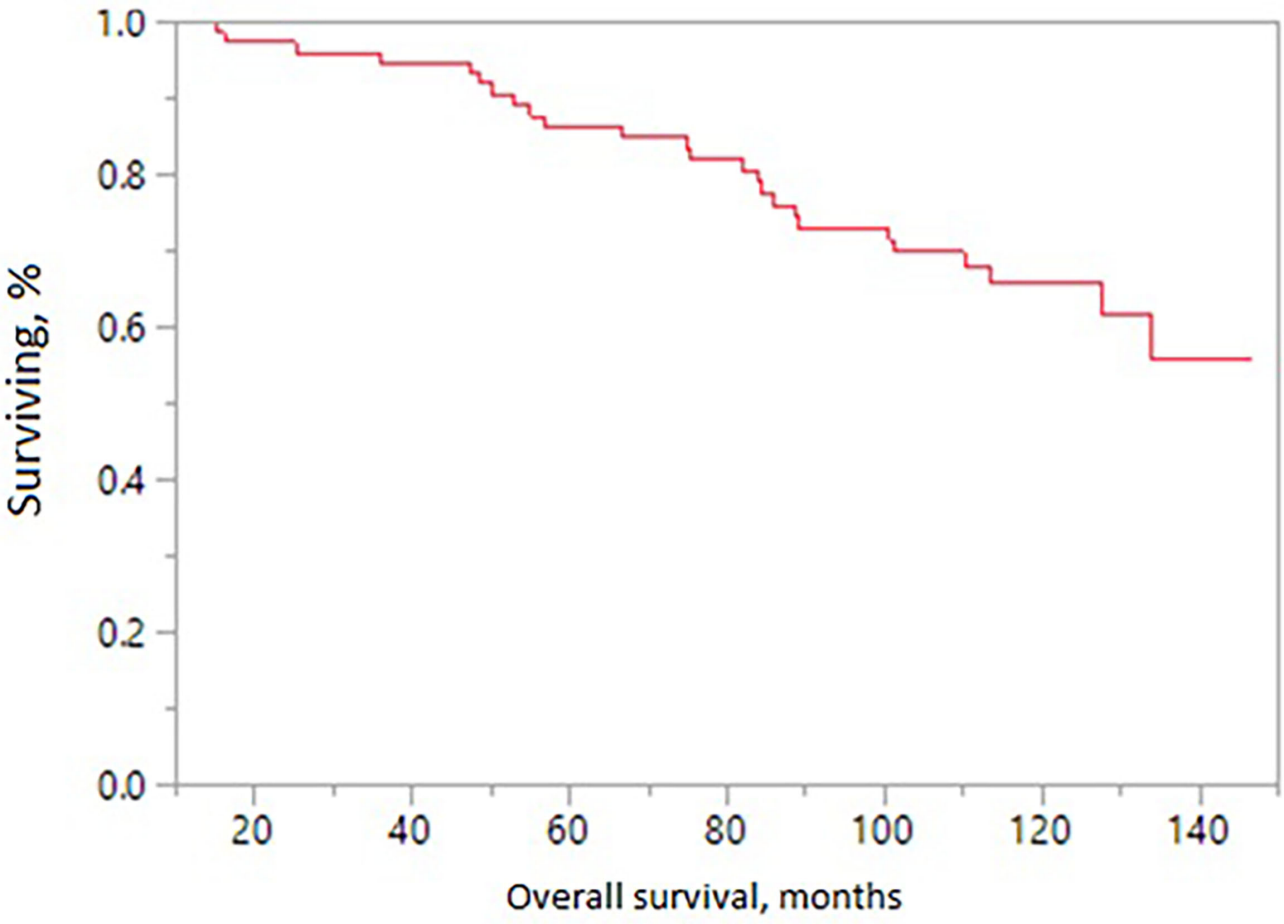
Overall survival in patients receiving MRI-directed boost.

**Figure 3:**
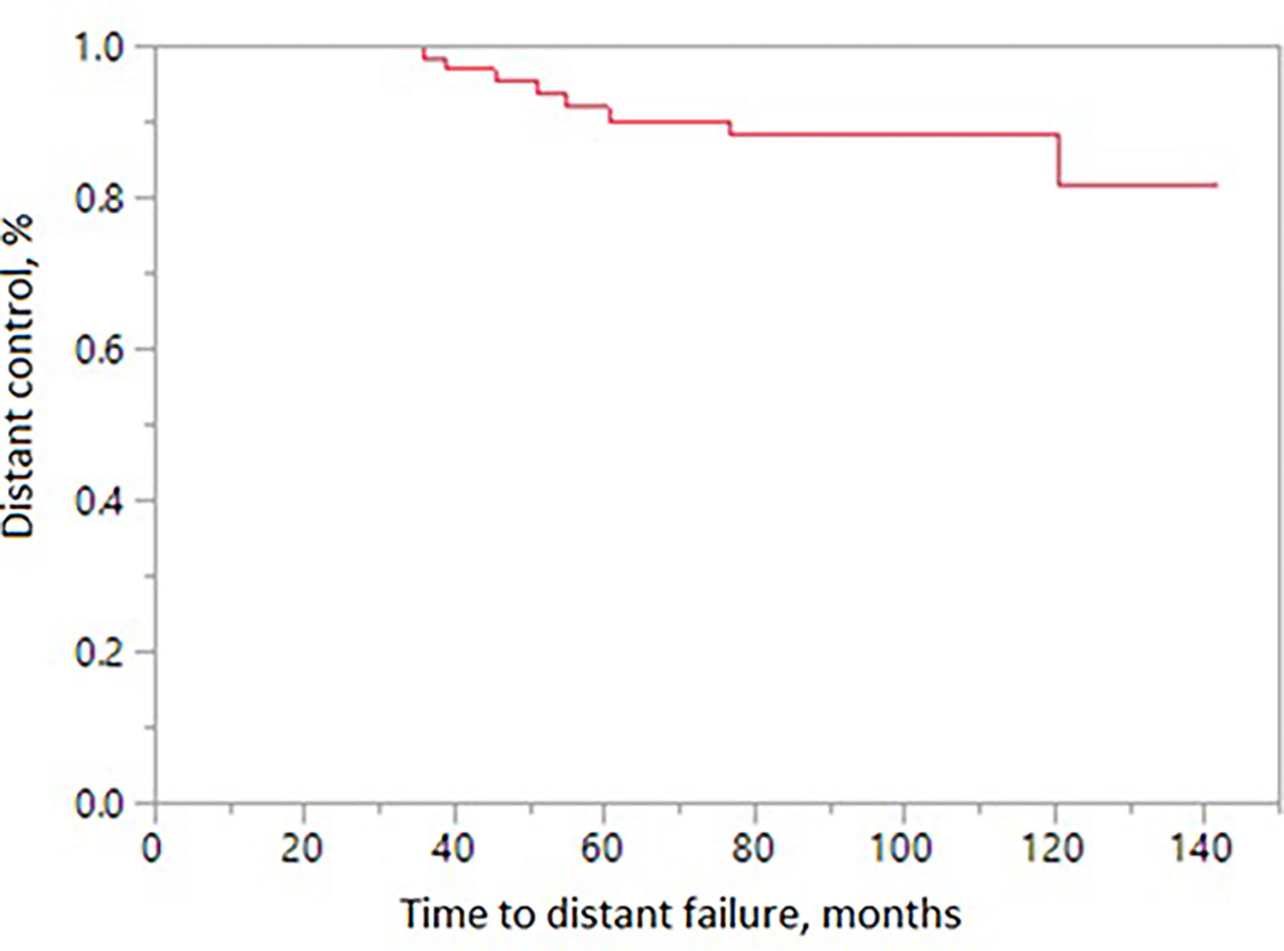
Distant control in patients receiving MRI-directed boost.

**Figure 4:**
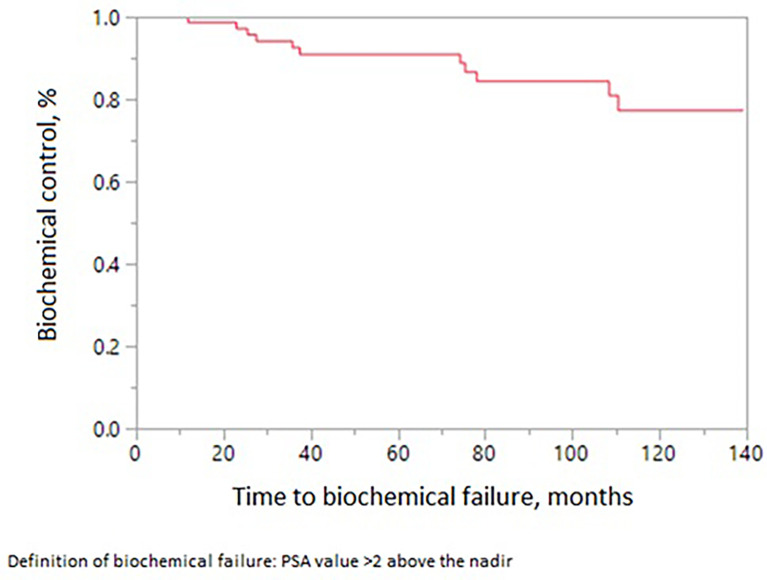
Biochemical control in patients receiving MRI-directed boost.

**Figure 5:**
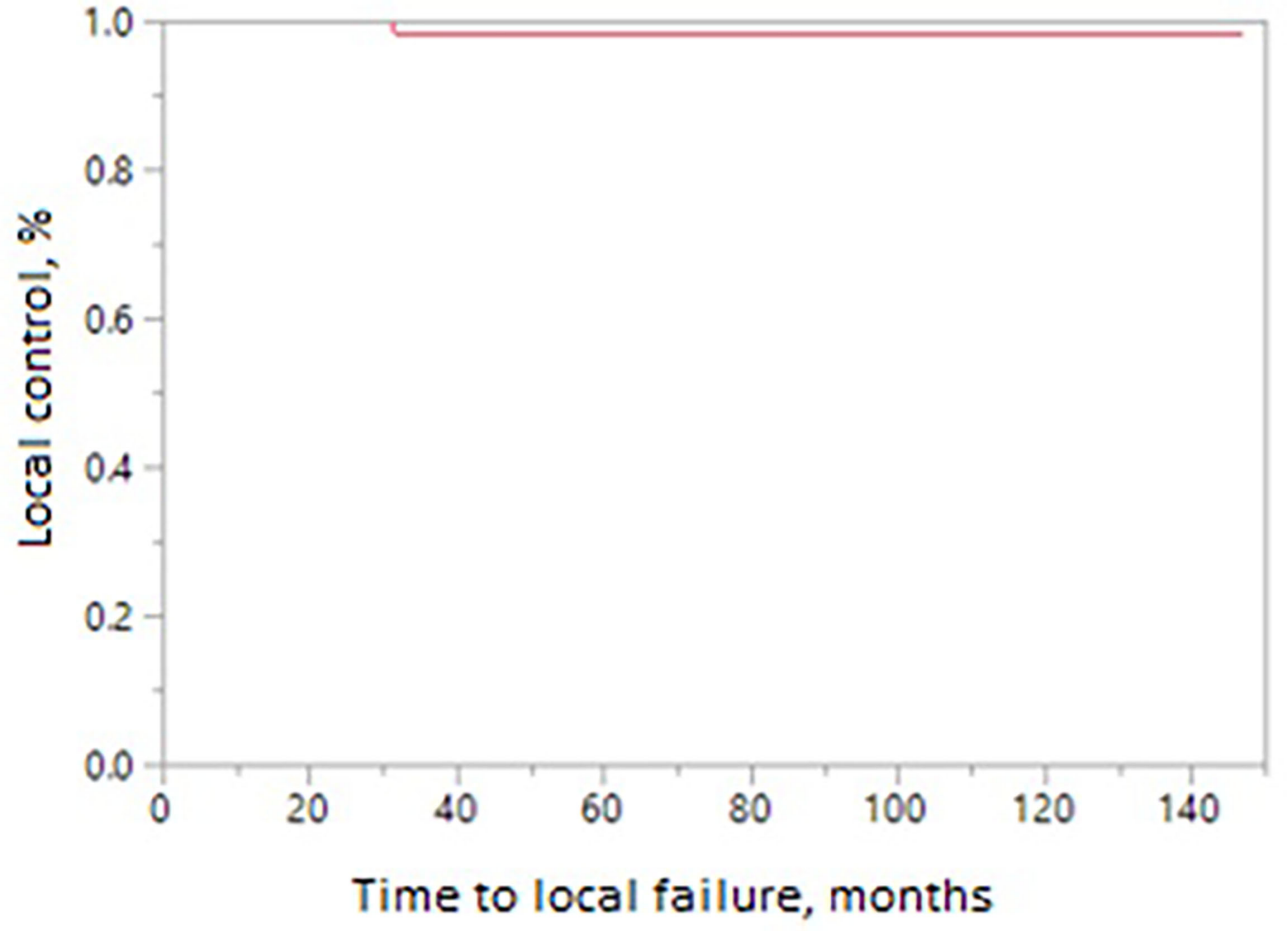
Local control in patients receiving MRI-directed boost.

The 10-year treatment outcomes are listed in **Table 1**. The 10-year biochemical control rates for low-risk, intermediate-risk, and high-risk diseases were 94%, 81%, and 88%, respectively (p = 0.35, **Figure 1**). The 10-year rates of overall survival, distant control, biochemical control, and local control were 66%, 88%, 77%, and 99%, respectively (**Figures 2**–**5**). Of the 25 patients who died, only four (5%) patients died because of their prostate cancer. The most common cause of death in the remaining patients was cardiovascular (16/21, 76%). On univariate analysis, a high T category and an elevated PSA level were associated with distant failure rate (p = 0.02, **Table 1**). Additionally, a high T category was associated with biochemical failure (p =0.004, **Table 1**), and an elevated PSA level was associated with local failure (p = 0.04, **Table 1**). GI and GU toxicity is described in detail in **Table 2**. There was no grade >3 GU and GI toxicities persisting at last follow-up. One (1%) patient had grade 2 GI toxicity at last follow-up.

**Table 2:** Acute and late-term GI and GU toxicity.

	Grade 0	Grade1	Grade 2	Grade 3
	n (%)	n (%)	n (%)	n (%)
Maximum acute GU toxicity	10 (13)	26 (33)	42 (54)	0
Maximum chronic GU toxicity	44 (56)	12 (15)	20 (26)	2 (3)
GU toxicity at last follow-up	58 (74)	4 (5)	16 (21)	0
Maximum acute GI toxicity	22 (28)	40(51)	16 (21)	0
Maximum chronic GI toxicity	66 (84)	9 (12)	3 (4)	0
GI toxicity at last follow-up	74 (95)	3 (4)	1 (1)	0

## Discussion

Here, we report our long-term outcomes using standard treatment of 77.4 Gy to localized prostate cancer with focal boost to intraprostatic lesion, identified on multiparametric MRI, to 81–83 Gy. Previously, we reported the feasibility of this approach with 3-year biochemical control, local control, distant control, and overall survival rates of 92%, 98, 95%, and 95% respectively (21). However, while promising, long-term results are important to report in terms of insuring both safety and efficacy.

Multiple randomized trials have shown that dose-escalated RT leads to improved biochemical control of prostate cancer (**Table 3**) (1–3, 6–8). RTOG 0126 randomized patients with intermediate-risk prostate cancer to conventionally fractionated radiotherapy (70.2 Gy) versus dose escalated radiotherapy (79.2 Gy) (8). They demonstrated that there was no difference in overall survival in the dose-escalated arm versus standard arm at 8 years (76% vs. 75%, p = 0.980) (8). However, there was an improvement in distant metastatic failure (4% vs. 6%, p = 0.05) and ASTRO Phoenix biochemical failure rates at 5 and 8 years (31% and 20% with 79.2 Gy and 47% and 35% with 70.2 Gy, p < 0.001). M.D Anderson performed a phase III clinical trial randomizing patients with prostate cancer to 70 Gy in 35 fractions versus 78 Gy in 39 fractions of photon radiation therapy using a four-field box 3D technique without hormone deprivation therapy (22). At 10 years, Kuban et al. reported a significant improvement in freedom from biochemical or clinical failure with the 78-Gy arm (73% vs. 50%, p = 0.004) (23). However, 10-year rates of grade 2 or higher gastrointestinal toxicity were twice as high in the 78-Gy arm (26% vs. 13%, p = 0.013), although grade 2 or higher genitourinary toxicity rates were not significantly different (13% vs. 8%) (23). In the long-term analysis of their dose escalation trial, Pasalic et al. reported a 20-year freedom from failure of 88% with the 78-Gy dose-escalated arm versus 82% in the 70-Gy standard conventional dose fractionation arm (p < 0.04) (22, 23).

**Table 3:** Important trials investigating dose escalation and hypofractionation for prostate cancer.

	*RTOG* 0126 (8)	M.D Anderson (3, 22, 23)	*CHHiP* (36)	*RTOG* 0415 (42)
Number of patients	748	301	3216	1115
Risk groups	Intermediate risk	Low risk, intermediate risk, high risk	Low risk, intermediate risk, high risk	Low risk
Arm 1	70.2 Gy/39 fx	70 Gy	74 Gy/37 fx	73.8 Gy/41 fx
Arm 2	79.2 Gy/44 fx	78 Gy	60 Gy/20 fx	70 Gy/28 fx
Arm 3	N/A	N/A	57 Gy/19 fx	Not applicable
OS	8-year: (75 vs. 76%, p =0.980).(8)	8-years 78% vs. 79%	5-year: 92.8% vs. 94.7% vs. 93.9%	
BC	8-year: 65% vs. 80%	10-year freedom from failure (FFF): 50% vs. 73% 15-year FFF: 81% vs. 88% 20-year FFF: 81% vs. 88%	5-year: 88.3% vs. 90.6% vs. 85.9%	7-year DFS: 75.6% vs. 81.8%
DC	8-year: (4% vs. 6%, p = 0.05		5-year: 97.5% vs. 97.9% vs. 97.4%	
Maximum chronic GU grade >=2 toxicity	7% vs. 12%	9.3% vs. 10.6%	9.2% vs. 11.7% vs. 6.6%	Not available
Maximum chronic GI grade >=2 toxicity	15% vs. 21%	11.4% vs. 25.2%	13.7% vs. 12% vs. 11%	Not available

There have been several retrospective and phase II studies which demonstrated the feasibility of simultaneous integrated boost for prostate cancer (**Table 4**) (14, 24–28). A systematic review by Feutren et al. demonstrated that focal intraprostatic lesion boost was associated with a 5-year biochemical control of 85% for a cohort of 812 patients (29). In their prospective trial, Wong et al. and Schild et al. showed the safety and efficacy of an image-guided boost using ProstaScint (19, 30). Using ProstaScint to delineate the IPL, IMRT was utilized to deliver 75.6 Gy in 42 fractions to the prostate and seminal vesicles and 82 Gy as a SIB to the IPL (19, 30). In their cohort of 71 patients, long-term 10-year biochemical control and overall survival was 85% and 69%, respectively. Our initial study showed favorable outcomes comparable to this prospective trial with a 10-year biochemical control of 77% and a 10-year survival rate of 66%. The projected life expectancy for a 76-year-old man in the US is 10.7 years (31). The 10.7-year survival rate of our patients was 62%, which appears better than expected. This may be in part a reflection of the fact that only four (5%) patients died due to prostate cancer.

**Table 4:** Important trials investigating boost for prostate cancer.

	*Prostacint/Wong (19, 30)*	*ASCENDE-RT (32, 33)*	*FLAME (34)*
Number of patients	71	398	571
Risk groups	Low, intermediate, and high risk	Intermediate and high risk	Intermediate and high risk
Arm 1	Initial: 75.6 Gy/42 fx Boost: 82 Gy	Initial 46 Gy, EBRT boost to 78 Gy	77 Gy/35 fx
Arm 2	N/A	125-Iodine LDR brachytherapy boost of 115 Gy	95 Gy/35 fx (SIB)
OS	5-year: 93% 10-year: 69%	5-year: 89% vs. 91% 7-year: 82% vs. 86% 9-year: 74%s. 78% v, (p > 0.05)	5-year: 91% vs. 88%, p = 0.50
BC	5-year: 94% 10-year: 85%	5-year: 84% vs. 89% 7-year: 75% vs. 86% 9-year: 62% vs. 83%, p <.001	5-year: 85% vs. 92%
DC	5-year: 97% 10-year: 91%	5-year: 93%, vs. 93% 7-year: 93% vs. 91% 9-year: 85% vs. 89% p > 0.05	5-year: 88% vs. 92%
Maximum chronic GU grade >=2 toxicity	39%	26.4% vs. 53.3%	23% vs. 27.8%
Maximum chronic GI grade >=2 toxicity	21%	23.4% vs. 40.4%	12.2% vs. 12.7%

The benefits of a boost with brachytherapy has been well documented (32, 33). In ASCENDE-RT, intermediate- and high-risk prostate cancer patients who received an initial 46 Gy to the pelvis were randomized to dose-escalated EBRT boost to 78 Gy versus LDR prostate brachytherapy boost (32). In comparison to men receiving 78 Gy EBRT, men receiving an LDR prostate brachytherapy boost had significantly higher rates of biochemical PFS at 5, 7, and 9 years (89%, 86%, and 83% for LDR boost vs. 84%, 75%, and 62% for EBRT, log rank p < 0.001.) (32) At 5 years, there was a significantly higher incidence of grade 3 genitourinary events with LDR prostate boost vs. dose-escalated EBRT (18.4% vs. 5.2%, p < 0.001) and no difference in grade 3 GI toxicity (8.1% vs. 3.2%, p = 0.124). Patients who received 78-Gy EBRT had twice the rate of biochemical failure compared to patients receiving an LDR brachytherapy boost (32).

While there have been retrospective studies that assess the feasibility and safety of simultaneous integrated external beam radiation boost to intraprostatic lesions, the FLAME trial was the first phase III clinical trial to assess the addition of a focal boost on rates of biochemical relapse and other endpoints (34). The FLAME trial was a phase III randomized trial which randomized 571 patients with intermediate-risk and high-risk prostate cancer to a standard treatment arm of 77 Gy to the prostate or boost arm with additional simultaneous boost of up to 95 Gy (34). In the conventional arm, there was a difference in 5-year biochemical disease-free survival favoring the focal boost arm (92% vs. 85%, p < 0.001) (34). Additionally, they demonstrated that biochemical disease-free survival and disease-free survival were statistically improved in the focal boost arm up to 7 years (p <.001) (34). Several differences are worth noting between our study and the FLAME trial. In the FLAME trial, high-risk and intermediate-risk patients made up 99% of the cohort. In contrast, our study had 77% intermediate-risk and high-risk patients, with 55% classified as intermediate risk. An overall comparison of outcomes between the two studies is challenging because there the FLAME study had approximately 84% high-risk patients vs. 22% within our study. Patients in the FLAME trial were treated with less fractions (77 Gy in 35 fractions) and with a focal boost to a higher dose (95 Gy). This equated to an equivalent total dose in 2-Gy fractions (EQD2) of 115.8 Gy with an α/β of 1.2 (34). In comparison, the EQD2 in our study with the same α/β used is 82 Gy. Although rates of biochemical control are similar, a higher EQD2 as used in the FLAME trial may be more effective for patients with a high-risk disease. Rates of late genitourinary and GI grade =2 toxicity were 23% and 12% for conventional fractionation versus 28% and 13% in the focal boost arm, which were not statistically significantly different. In our patient cohort, we reported a similar chronic GU grade =;2 toxicity of 29% but a lower GI grade =2 toxicity of 4%, respectively. By last follow-up evaluation, the GU and GI grade =2 toxicity was 21% and 1%, respectively. The lower rates of chronic grade =2 GI toxicity (chronic 4% and 1% at last follow-up) may be attributable to a lower EQD2 (82 Gy) in our study versus the FLAME trial (115.8 Gy). Our long-term results show that MRI-guided boost to prostate cancer lesions is feasible and associated with low long-term morbidity.

Recently, there has been a shift in the radiation treatment paradigm for prostate cancer toward hypofractionation (**Table 3**). In RTOG 0415, patients with low-risk prostate cancer were randomized to 73.8 Gy/41 fractions versus 70 Gy/28 fractions, with non-inferior DFS (85% vs. 86% with HR 0.85) (35). Late grade 2 and 3 gastrointestinal toxicities were increased in patients who received hypofractionation (36). Similarly, the CHHiP trial randomized patients with localized prostate cancer to three arms: 74 Gy/37 fractions, 60 Gy/20 fractions, and 57 Gy/19 fractions (37). The moderately hypofractionated 60 Gy in 20 fractions was shown to have a non-inferior biochemical failure rate of 90.6% vs. 88.3% (37). There were no difference in late GI or GU toxicity between conventional fractionation versus hypofractionation in this study.

The results of the FLAME and ProstaScint trials support the continued efforts to use advanced technologies to deliver focal boosts to prostate tumors. The development of prostate-specific membrane antigen (PSMA)-targeted imaging provides new opportunities to safely boost prostate tumors. Although multiparametric MRI is consistently used to identify prostate cancer given its high sensitivity, specificity, and predictive value, it is possibly advantageous to use the newest imaging when designing radiation boost techniques (38, 39). Multiple studies have demonstrated that PSMA PET scan and mpMRI have similar sensitivity and specificity for identifying local diseases (39). However, there are mixed data regarding whether there is non-inferior sensitivity and specificity for identifying seminal vesicle invasion or extracapsular extension (40). On the other hand, PSMA is effective in identifying metastatic diseases which can also be targeted and treated. In the oligometastatic setting, the SABR-Comet trial established that the utilization of stereotactic body radiotherapy vs. palliative radiation provides significantly improved overall survival at 5 years (42.3% vs. 17.7%, p = 0.006) (41). The STAMPEDE trial evaluated radiotherapy to the prostate in men with newly diagnosed metastatic prostate cancer; radiation to the prostate in men with a low metastatic disease was found to confer a survival benefit (3 year OS 81% with RT vs. 73% control, p = 0.007) (42).

This treatment approach was initiated to increase the therapeutic ratio of radiation treatment to prostate cancer but does have limitations. It is limited by its retrospective nature and by the fact that most patients had low-risk and intermediate-risk diseases (80%). The future applicability of this regimen is limited given the overall shift in practice and standard-of-care treatment toward hypofractionation. While our study demonstrates durable biochemical control and overall survival with low toxicity using a simultaneous integrated boost in the setting of conventional fractionation, more recent trials have altered the standard of care to hypofractionated courses of radiotherapy. Therefore, studies which focus on dose-escalated SIB in the setting of hypofractionation are needed. Currently, the HEIGHT trial is an ongoing interventional clinical trial investigating the delivery of hypofractionated boost to the dominant tumor lesion identified by multiparametric MRI. The lesion will be treated with an absolute dose of 89.3–91.2 Gy, while the prostate receives 76 Gy over 38 fractions. Questions regarding the feasibility, safety, and efficacy of MRI guided boost to tumors with hypofractionation will be answered.

In summary, our experience demonstrated that an MRI-guided focal boost to intraprostatic lesions produces durable long-term biochemical and local control without severe (grade 3 or greater) toxicity. MRI-guided boost to prostate lesions is an effective strategy to enhance biochemical control in patients. Our study provides validation that MRI-guided boost can be safely employed without increased risk of increased long-term toxicity. This was likely true because we left the normal tissue dose limitations in place when administering the boost doses. Given the evolution of prostate cancer treatment with shorter treatment courses, further studies evaluating dose-escalated boost in the setting of hypofractionation and stereotactic body radiotherapy are needed. The incorporated use of MP-MRI and PSMA PET-directed boost techniques with hypofractionation or stereotactic body radiotherapy should be investigated in future trials to potentially improve both disease control and treatment-related toxicities.

## Data availability statement

The raw data supporting the conclusions of this article will be made available by the authors, without undue reservation.

## Ethics statement

The studies involving human participants were reviewed and approved by Mayo Clinic Institutional Review Board. Written informed consent for participation was not required for this study in accordance with the national legislation and the institutional requirements.

## Author contributions

SS conceptualized the study and performed the formal statistical analysis. BL wrote this study. All authors were involved with review, editing, and final approval of this manuscript.

## Conflict of interest

SS is a writer and editor for UptoDate.

The remaining authors declare that the research was conducted in the absence of any commercial or financial relationships that could be construed as a potential conflict of interest.

## Publisher’s note

All claims expressed in this article are solely those of the authors and do not necessarily represent those of their affiliated organizations, or those of the publisher, the editors and the reviewers. Any product that may be evaluated in this article, or claim that may be made by its manufacturer, is not guaranteed or endorsed by the publisher.
